# Transfer Across Different Machines by Transfer Function Estimation

**DOI:** 10.3389/frai.2022.811073

**Published:** 2022-03-02

**Authors:** Omri Matania, Renata Klein, Jacob Bortman

**Affiliations:** PHM Laboratory, Department of Mechanical Engineering, Ben-Gurion University of the Negev, Beer Sheva, Israel

**Keywords:** zeros and poles, adaptive clutter separation (ACS), transfer function estimation, minimum phase, autoregressive moving-average (ARMA) model, transfer across different machines (TDM)

## Abstract

A digital twin is a promising evolving tool for prognostic health monitoring. However, in rotating machinery, the transfer function between the rotating components and the sensor distorts the vibration signal, hence, complicating the ability to apply a digital twin to new systems. This paper demonstrates the importance of estimating the transfer function for a successful transfer across different machines (TDM). Furthermore, there are few algorithms in the literature for transfer function estimation. The current algorithms can estimate the magnitude of the transfer function without its original phase. In this study, a new approach is presented that enables the estimation of the transfer function with its phase for a gear signal. The performance of the new algorithm is demonstrated by measured signals and by a simulated transfer function.

## Introduction

Prognostic health monitoring (PHM) by vibration signal analysis is a widespread method for condition-based maintenance (Carden and Fanning, 2004; Randall, 2004a,b, 2011). The vibration signals of the machinery are measured *via* acceleration sensors and are processed and analyzed by signal processing (Randall et al., 2012; Gousseau et al., 2016; Peeters et al., 2017) and machine learning algorithms (Abu-Mahfouz, 2005; Lei, 2016; Zhang et al., 2019; Lei et al., 2020). The revival of neural networks in the last decade *via* the incarnation of deep learning (Goodfellow et al., 2016) has boosted the abilities of machine learning algorithms to facilitate PHM by vibration analysis (Lei, 2016; Lei et al., 2020).

**Figure 1:**
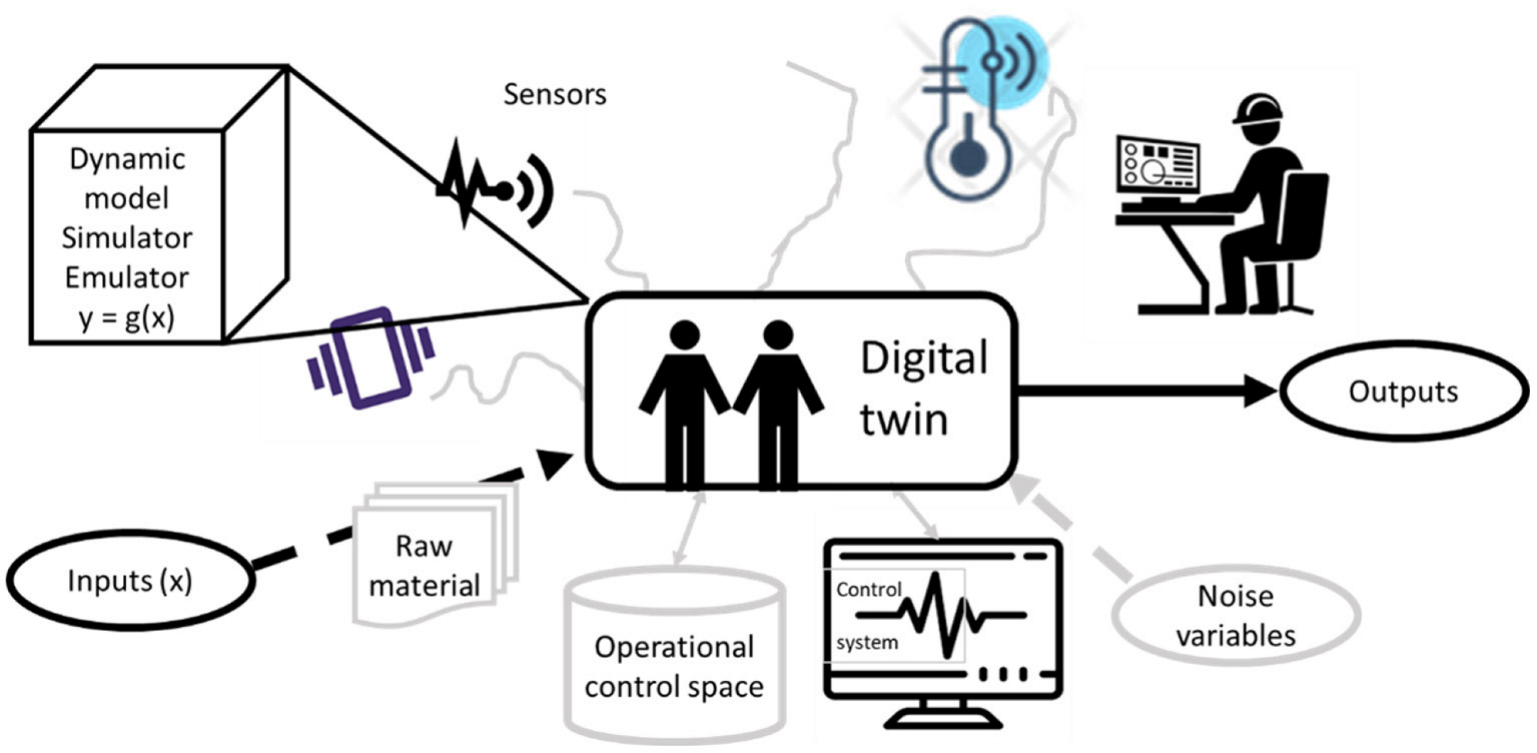
Representation of the behavior of a digital twin and the use of emulators of the physical system. The picture was reproduced from Kenett and Bortman (2021).

As explained in Lei et al. (2020), two main challenges hamper the ability to apply state-of-the-art deep models in PHM: (1) the shortcomings in signal examples with faults in real machinery and (2) the ability to generalize across different machinery. A digital twin is an important novel tool (Kenett and Bortman, 2021) that can mitigate the first challenge: it enables the behavior of fault signals to be learned by several examples and it can generate many synthetic new examples. As explained in Kenett and Bortman (2021), the digital twin can learn from a wide source of inputs (as illustrated in Figure 1), and it generates more accurate synthetic data as it accumulates more data.

Several real-world examples have proved the concept and the utility of digital twins: in Xia et al. (2021), a sparse de-noising auto-encoder was trained using data generated from a digital twin, enabling diagnosis of a fault in a triplex pump; in Xu et al. (2019), data generated from a digital twin helped to train a deep neural network to diagnose faults in a car body-side production line; and in Jain et al. (2020), data generated from a digital twin was used to diagnose faults in distributed photovoltaic systems. There are many other real-world examples in which data generated from a digital twin was used to train a learner to automatically diagnose faults; more examples and information can be found in Kenett and Bortman (2021).

The second challenge—the ability to generalize across different machinery—can prevent successful application of a digital twin if there are shortcomings in fault signal examples from the new machine. As explained by Lei et al. (2020), some machine learning algorithms (including algorithms for gear diagnostics) obtain satisfactory results on some problems, but these are mostly based on the impractical assumption (Yang et al., 2019) that the labeled data are large enough and contain faulty data besides healthy data, thus, adequately representing the data distribution under different fault conditions (Guo et al., 2019). As stated in Lei et al. (2020), this assumption is not practical as it is difficult to collect labeled faulty data for two reasons: (1) machines work most of the time under a healthy state, while faults are rare; hence, healthy data are much more common than faulty data. (2) It is hard to label data because it is very expensive to stop a machine to inspect its rotating component status (perhaps requiring a long and expensive dismantling of the machine and its components) and label the data. One possibility to circumvent this challenge is to learn from one machinery and generalize the data to a new one by transfer across different machines (TDM) (Lei et al., 2020). A major challenge of TDM in the current study was to mitigate the effects of the transfer function on the signal resulting from the transfer across different machines.

The main and inherited variation across different machines or across different sensors in the same machine is the transfer function between the rotating components and the sensor (Randall, 2011; Madar et al., 2019; Dadon et al., 2020). Because the transfer function distorts the signal's shape, this complicates the ability to generalize across different machines.

**Figure 2:**
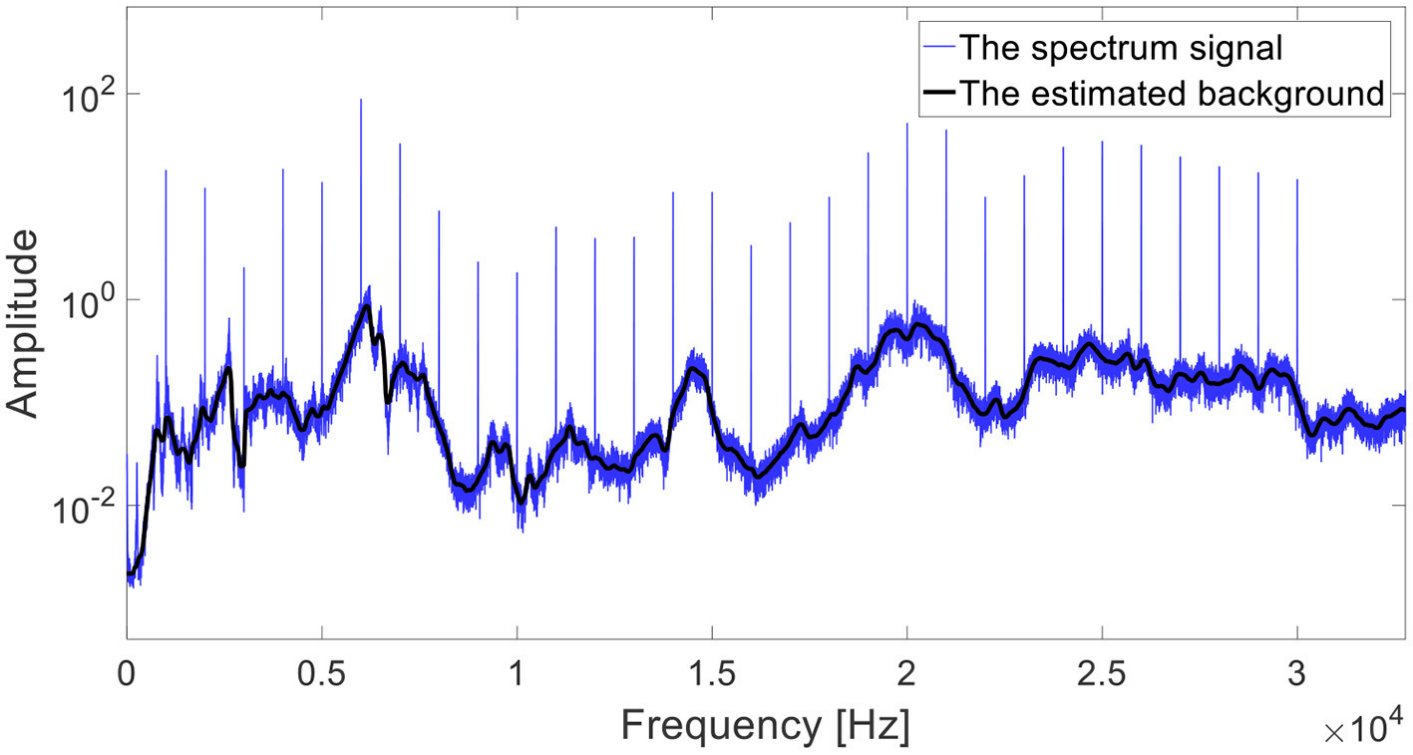
An example of an estimated spectrum background (black) of a spectrum signal (blue).

The spectrum of rotating machinery contains discrete frequencies of its rotating components (such as bearings, gears, etc.) and the background spectrum, associated with the magnitude of the transfer function, as depicted in Figure 2 (Randall, 2011; Borghesani et al., 2013; Klein, 2017). As a result of the wideband noise induced by the surface shape (Dadon et al., 2020) and the transmission errors of gears (Randall, 2011; Lu et al., 2022), it can be assumed that the magnitude of the transfer function of gears can be approximated by the background. As explained in Matania et al. (2021), the spectrum background of a vibration signal can be estimated by several techniques such as an autoregressive (AR) model (Sawalhi and Randall, 2005, 2013; Randall, 2011), cepstrum liftering (Childers et al., 1977; Peeters et al., 2016; Smith and Randall, 2016; Randall, 2017; Matania et al., 2021), and adaptive clutter separation (ACS, Klein, 2017; Matania et al., 2021, 2022).

Although, for gears, the magnitude of the transfer function can be approximated by the background, the phase cannot be approximated directly by the spectrum signal. Currently, to mitigate this challenge, it is assumed that the transfer function holds the minimum phase assumption, which states that besides the poles, the zeros of the transfer function are inside the unit circle (Oppenheim et al., 1999; Randall, 2011) (the poles hold this property because the transfer function must be stable). However, as demonstrated in Section Theoretical Background, the minimum phase assumption does not hold for some transfer functions.

This study demonstrates the importance of transfer function estimation for transfer across different machines (TDM) for enhancing the generalization abilities of digital twins to new systems. It presents a new approach for estimating the phase of transfer functions (without assuming that the phase is a minimum one) and demonstrates how this new technique improves the generalization across different machines.

Current methods are described in Section Theoretical Background. The new approach is presented in Section Non-minimum Phase Estimation, and applied in Section Demonstration on Measured Signals and Simulated Transfer Function for TDM in order to improve the generalization of convolutional neural networks (CNNs).

## Theoretical Background

The estimation of the transfer function for TDM (Figure 6) is composed of four procedures described in the literature. These procedures are explained in the sections that follow.

### Adaptive Clutter Separation

**Figure 3:**
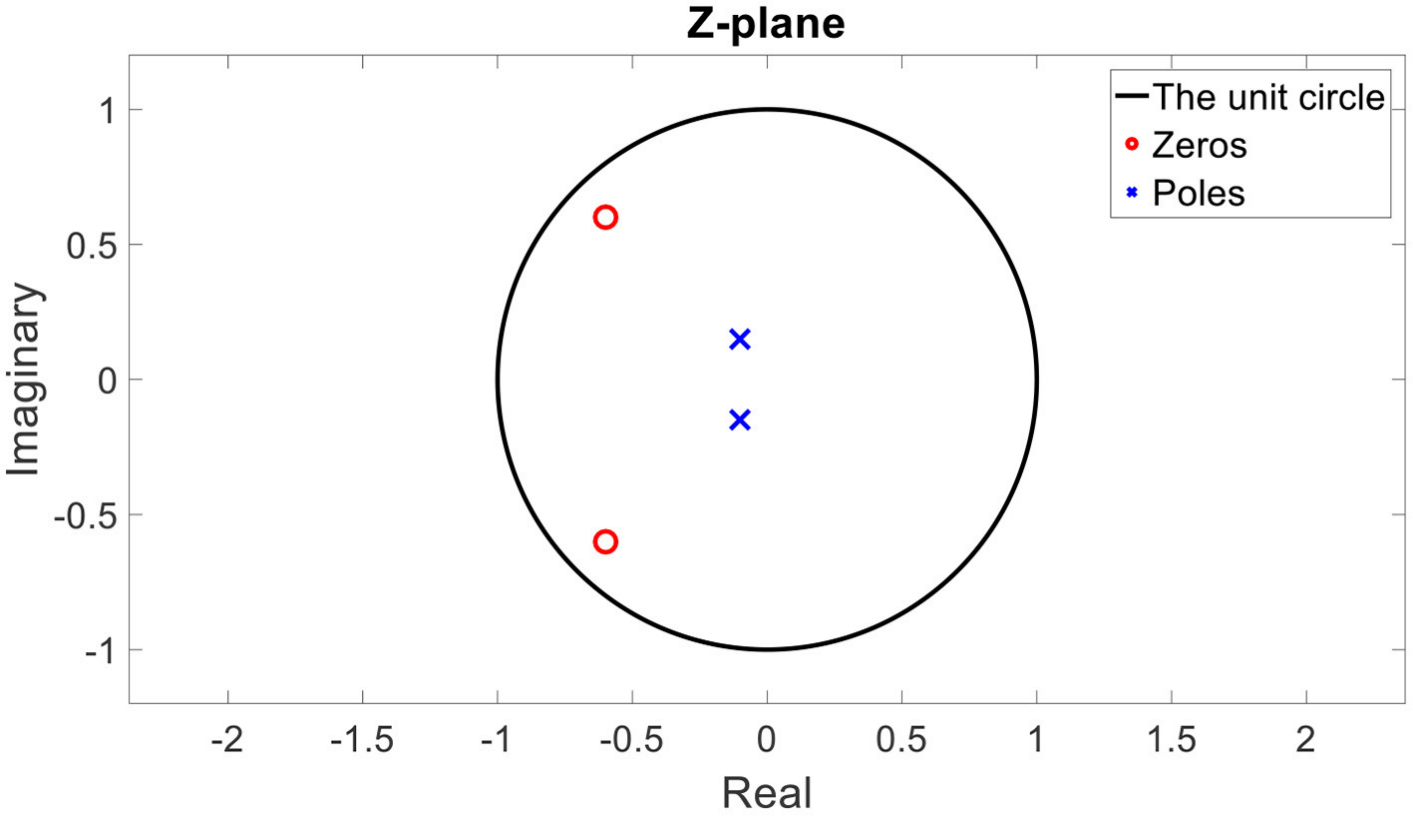
An example of the poles and zeros of a minimum phase transfer function in a Z-plane (after Z transform).

ACS is an algorithm for background estimation, first presented in Klein (2017), and widely investigated for stationary cases in Matania et al. (2021) and for non-stationary cases in Matania et al. (2022). It estimates the spectrum background by filtering out extreme deviations. As described in Matania et al. (2021) in Figure 3, in the first step, the algorithm separates the spectrum into consecutive segments, and in the second step, it selects the median in each of them. In the third (and last) step, the algorithm interpolates the selected values by a piecewise cubic Hermite interpolating polynomial (PCHIP). An example of an estimation of background by ACS is presented in Figure 2 by a black line.

As explained in Matania et al. (2021), ACS and cepstrum liftering are preferable over AR for background estimation. In the current study ACS is applied, but cepstrum liftering can also be applied, as it will show similar results.

### The Minimum Phase Assumption

The poles of every stable transfer function are inside the unit circle after Z-transform (Oppenheim et al., 1999). Furthermore, if the inverse transfer function is also stable, its poles are also inside the unit circle; hence, the zeros of the original transfer function are also inside the unit circle. In Figure 3, an example of the positions of the poles and zeros in the Z-plane of a minimum phase transfer function are depicted.

For the minimum phase transfer function, the phase can be estimated from the magnitude of the transfer function in the cepstrum domain. As explained in Oppenheim et al. (1999) and Randall (2011), for estimation of the transfer function (magnitude and phase), the negative quefrencies of the magnitude of the transfer function are set to zero, and the positive quefrencies are doubled.

**Figure 4:**
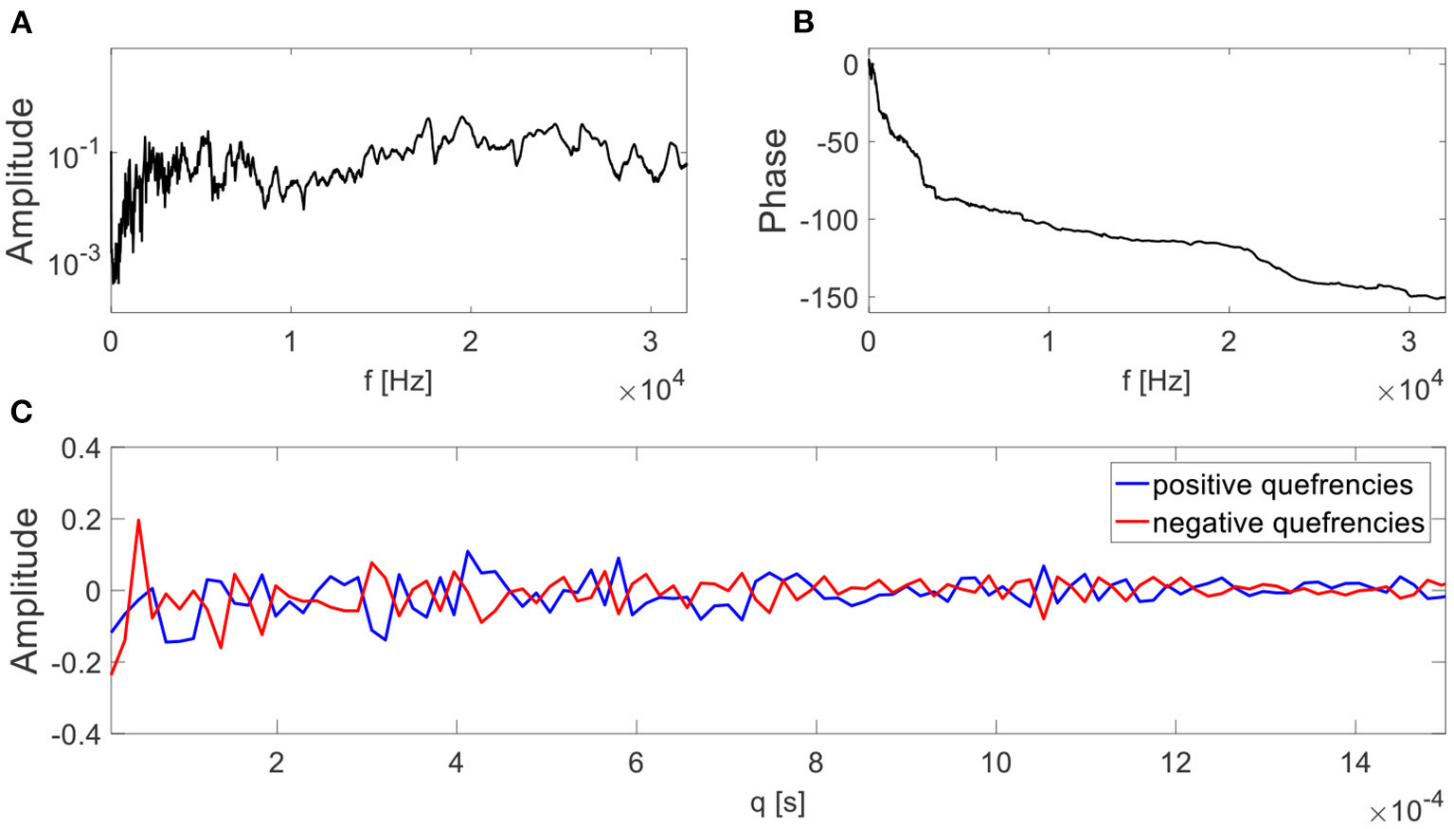
An example of a measured transfer function from Matania et al. (2021). The transfer function magnitude and phase are depicted in **(A,B)**, respectively. In **(C)**, the positive and negative quefrencies of the transfer function are presented on the same axis.

There are transfer functions that do not hold the minimum phase assumption. For example, in Matania et al. (2021), 24 transfer functions were measured by a hammer-tap experimental analysis on several test rigs (Cunha and Caetano, 2006). Those measured transfer functions are not minimum phase because their negative quefrencies are not equal to zero. An example is presented in Figure 4C, where the negative quefrencies are not equal to zero. The magnitude of the transfer function is presented in Figure 4A and its phase in Figure 4B.

### ARMA Model

The autoregressive moving-average (ARMA) model fits the noise behavior to Equation (1) (Choi, 1992; Chen et al., 1995). The ARMA model estimates the coefficients that best explain the statistical distribution of the noise [minimizing the mean squared error (MSE, Wallach and Goffinet, 1989; Lehmann and Casella, 2006; Shalev-Shwartz and Ben-David, 2013)] between the next sample and the predicted value based on the former samples. These coefficients correspond to the poles and zeros of the transfer function.


$$\begin{array}{l} a_{n}y[n]\;+\;a_{n\;-\;1}y[n\;-\;1]\;+\;\ldots \;+\;a_{n\;-\;k}y[n\;-\;k]\;=(1)\\ b_{n}x[n]\;+\;b_{n\;-\;1}x[n\;-\;1]\;+\;\ldots \;+\;b_{n\;-\;k}x[n\;-\;k] \end{array}$$

### Possible Positions of the Zeros

**Figure 5:**
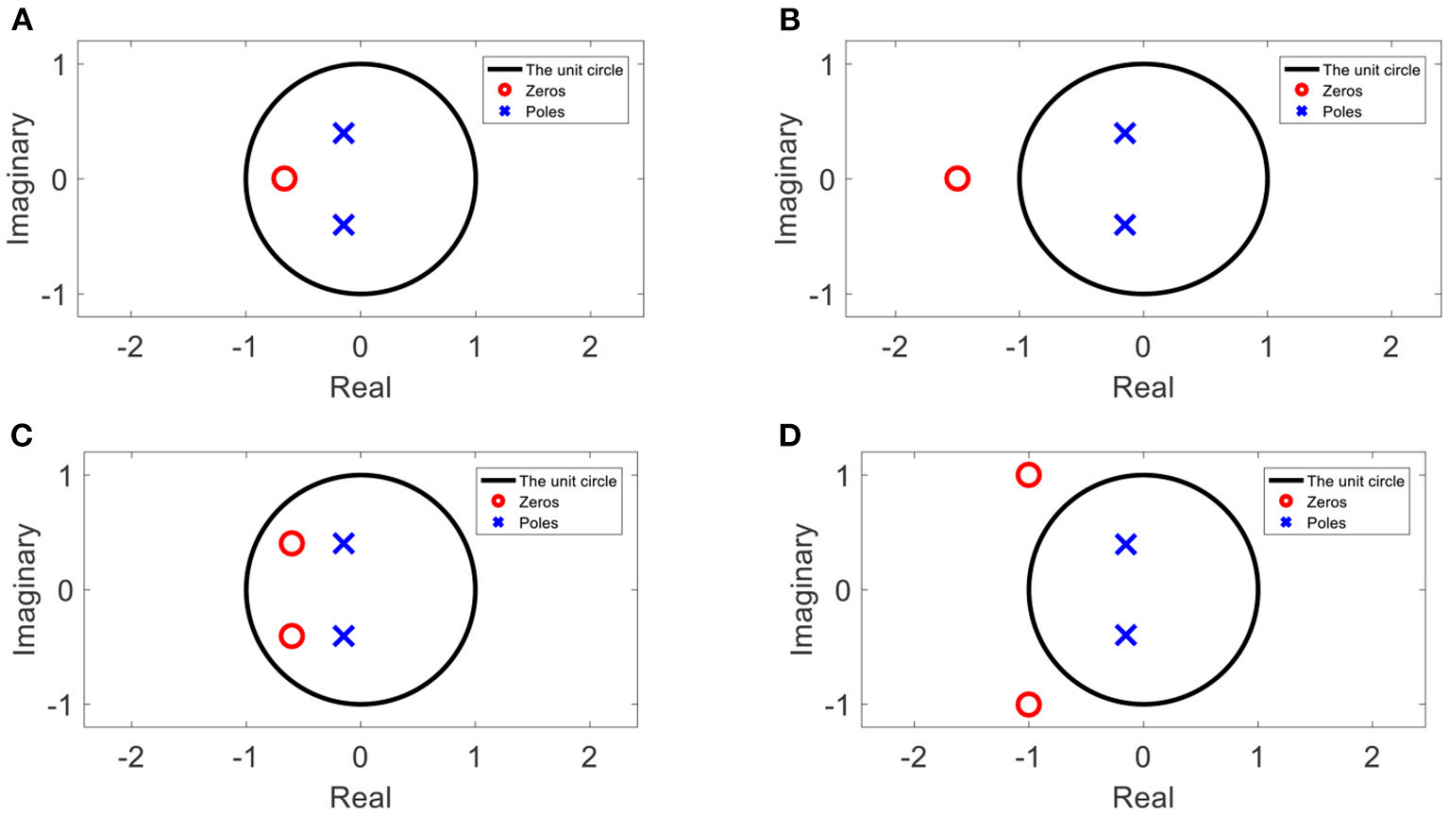
An example of inverse zeros with or without imaginary components. **(A)** A zero inside the unit circle, **(B)** the inverse proportion of the zero from **(A)**, **(C)** a pair of zeros inside the unit circle, and **(D)** the inverse proportion of the pair of zeros from **(C)**.

When the magnitude of the transfer function is known, but the phase is not the minimum phase, the phase cannot be estimated as described in Section The Minimum Phase Assumption. However, as explained in Oppenheim et al. (1999), the possible positions of the zeros are limited. For a real value zero (with an imaginary component equal to 0), its possible positions are inside the unit circle, as in the case of minimum phase (Figure 5A), or inverse proportional to that position in the unit circle (Figure 5B). For a zero with an imaginary component, its possible positions are inside the unit circle, together with its conjugate zero in the case of minimum phase (Figure 5C), or inverse proportional to the unit circle together with its conjugate zero (Figure 5D).

## Non-Minimum Phase Estimation

**Figure 6:**
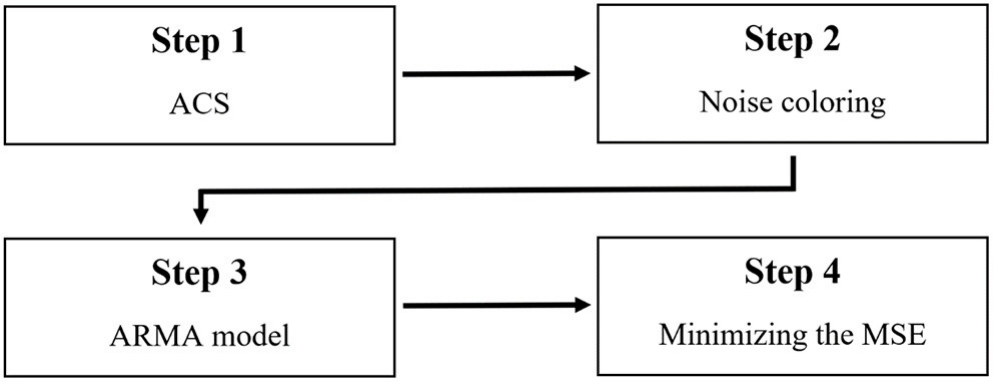
Block diagram of the new algorithm for estimating the transfer function phase without the minimum phase assumption.

The new algorithm estimates the transfer function—magnitude and phase—in four steps, as described in Figure 6.

1. In the first step, the magnitude of the transfer function is estimated based on the estimated background by ACS. The segment size can be set based on the selection mechanism described in Matania et al. (2021), in which the segment size is set by tuning the trade-off between a too large segment size causing an insufficient reconstruction of “fast variations” in the background shape and a too small segment size that fails to filter sharp picks.
2. In the second step, the estimated magnitude is multiplexed in the frequency domain with white noise (random Gaussian noise with a mean of zero and a variance of one), and the result is converted to the time domain.
3. In the third step, the poles and zeros of the transfer function are estimated by the ARMA model, as explained in Section ARMA Model. The number of poles and zeros can be set by minimizing the MSE between the estimated background by ACS and the estimated magnitude of the transfer function after the ARMA model. In the current article, we search over the number of poles of 0, 5, 10, and 15 and the number of zeros of 0, 5, 10, and 15.
4. In the fourth step, the phase is estimated by locating the zeros in all of their possible positions (i.e., by locating them in all the optional positions inside and outside the unit circle, as described in Section Possible Positions of the Zeros), and the phase that minimizes the MSE between the health signal in the first system and the healthy signal in the new system is selected.

The pole and zero degrees of the ARMA model can be set by searching the minimal degrees of the poles and the zeros such that the estimated magnitude of the transfer function fits the estimated background. The ACS parameters can be selected as explained in Matania et al. (2021) and Matania et al. (2022).

## Demonstration on Measured Signals and Simulated Transfer Function

In this paper, the dataset was generated by 300 measured signals with different fault sizes and a simulated transfer function. A CNN was designed to learn from the measured signals in the training phase and a healthy signal after the simulated transfer function, and to generalize in the test phase of measured signals after the simulated transfer function. In Section Dataset. the measured signals, the simulated transfer function, and the dataset are described. In Section Examination of the CNN, the abilities of the CNN to generalize to the new signals after the transfer function is examined.

### Dataset

**Figure 7:**
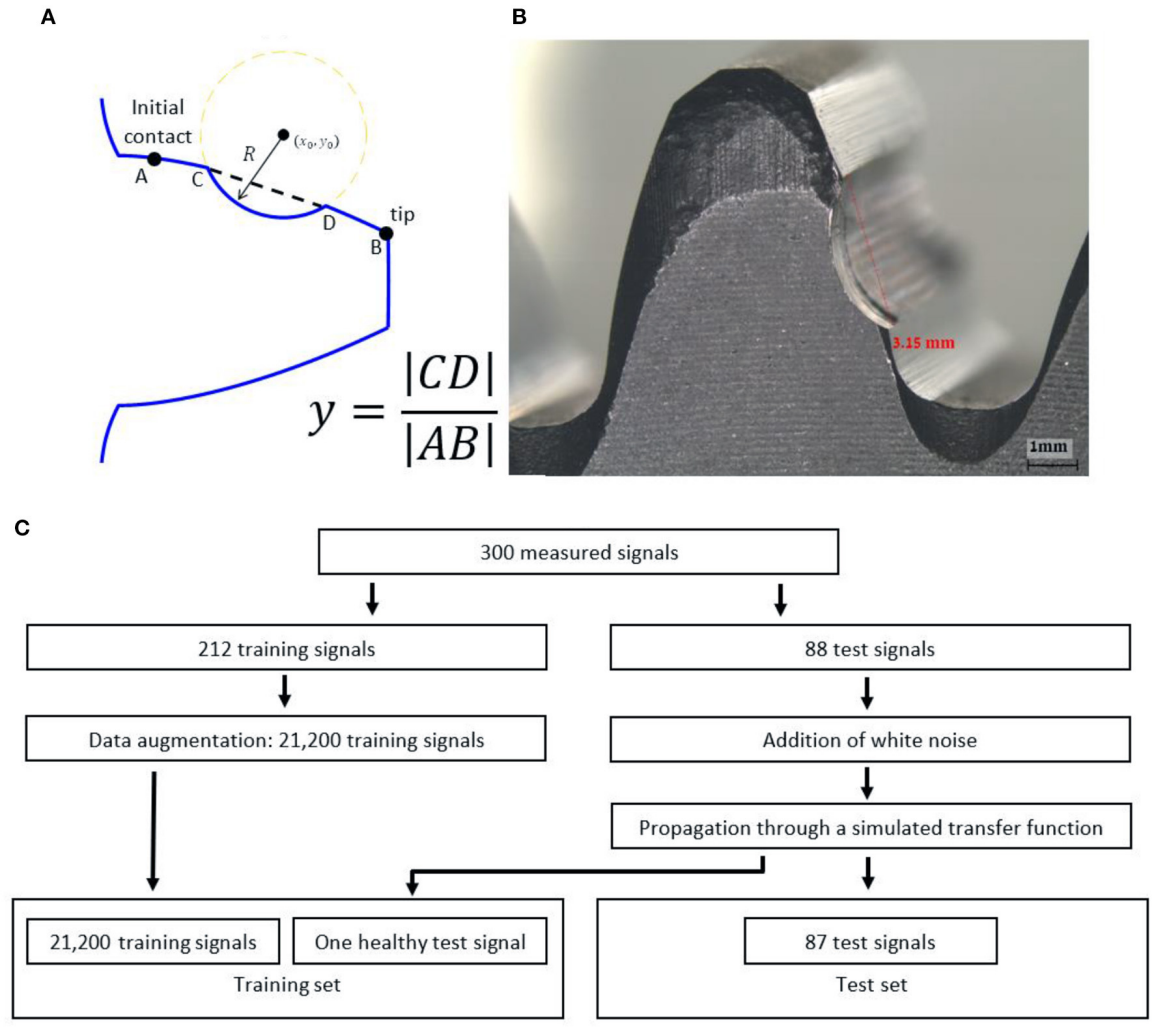
Description of the dataset and explanation of the fault size. **(A)** Explanation about the definition of the fault size. **(B)** An example of a FTFF of a gear. **(C)** The block diagram of the generation of the dataset. The data augmentation includes a random cyclic rotation of the synchronous average signal because the initial time of the signal was random.

The CNN was applied to measurements of a gearbox with several full tooth face faults (FTFF) on the gear, as depicted in Figure 7. The gearbox contains a gear with 38 teeth, a pinion with 17 teeth, module 3, a precision grade of AGMA 10, with revolutions per second (RPS) of 40 Hz and a load of 160 N. The sizes of the faults are presented in Table 1 and explained in Figure 7. The used signals were measured by an acceleration sensor composed on the test rig depicted in Figures 10, 11 in Dadon et al. (2020). The duration of each record was 60s, and six records were measured for each fault size. More details and descriptions of the experiment system can be found in Dadon et al. (2020).

**Table 1:** Table of fault sizes.

**Number of signals**	**Fault size**
60	0
60	0.31
60	0.49
60	0.75
60	0.85

In the current study, each record was divided into 10 consecutive signals with a duration of 10s, and the synchronous average was calculated for each signal.

**Figure 8:**
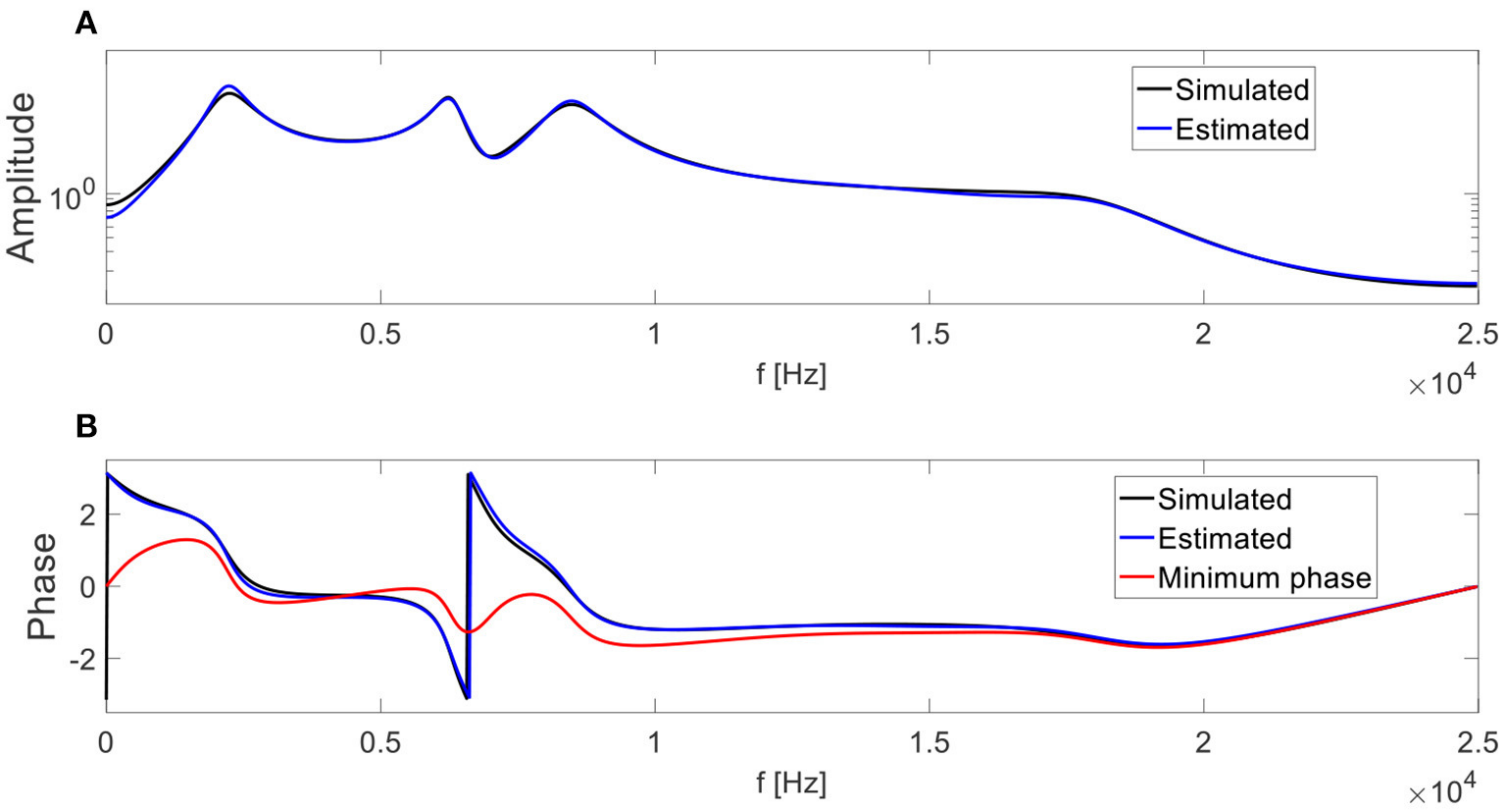
The simulated transfer function and its estimations by the new technique (blue) and using a minimum phase assumption (red). The graph of the estimated transfer function by the new technique conceals some of the segments of the simulated transfer function. **(A)** Magnitude of the transfer functions, and **(B)** phase of the transfer functions. The ACS segment size was 50 bins. The degrees of the coefficients of the poles and zeros in the ARMA model were 10 and 5, respectively.

An example of an FTFF is presented in Figure 7. The fault size is the value $y\;=\;\frac{\left|CD\right|}{\left|AB\right|}$. The simulated transfer function is also presented in Figure 8. The training set comprises the measured signals before the transfer function and a measured healthy signal with noise added after the transfer function, and the test set contains the measured signals with noise added after the transfer function. The noise was white noise with an RMS of 10% of the RMS of a healthy measured signal. The training set was augmented by random circular rotations of the vibration signals to improve generalization abilities (Goodfellow et al., 2016).

### Examination of the CNN

A CNN with seven layers was applied on the dataset:

1. The first layer was a convolutional layer with 32 kernels with length of 3 and ReLU activation.
2. The second layer was a max pool over two values.
3. The third layer was a convolutional layer with 32 kernels with length of 3 and ReLU activation.
4. The fourth layer was a max pool over two values.
5. The fifth layer was a flatten layer.
6. The sixth layer was a fully connected layer with 20 neurons and ReLU activation.
7. The seventh layer was a fully connected layer with one neuron and sigmoid activation.

The loss was the mean absolute error, the optimizer was Adam with a starting learning rate of 0.001, and the validation set contained 30% of the samples. The batch size was set to 32, and the number of epochs was set to 10 (using early stopping regulation, Goodfellow et al., 2016).

The CNN was applied three times under three different pre-processing steps:

1. Pre-processing A: the training set was inserted into the CNN as is.
2. Pre-processing B: the training set was multiplied by the estimated transfer function under the minimum phase assumption.
3. Pre-processing C: the training set was multiplied by the estimated transfer function with the estimated phase by the new technique.

The transfer function magnitude was estimated, together with its phase based on the healthy signal in the training set, originally from the test set. The estimated transfer function is depicted in Figure 8.

**Figure 9:**
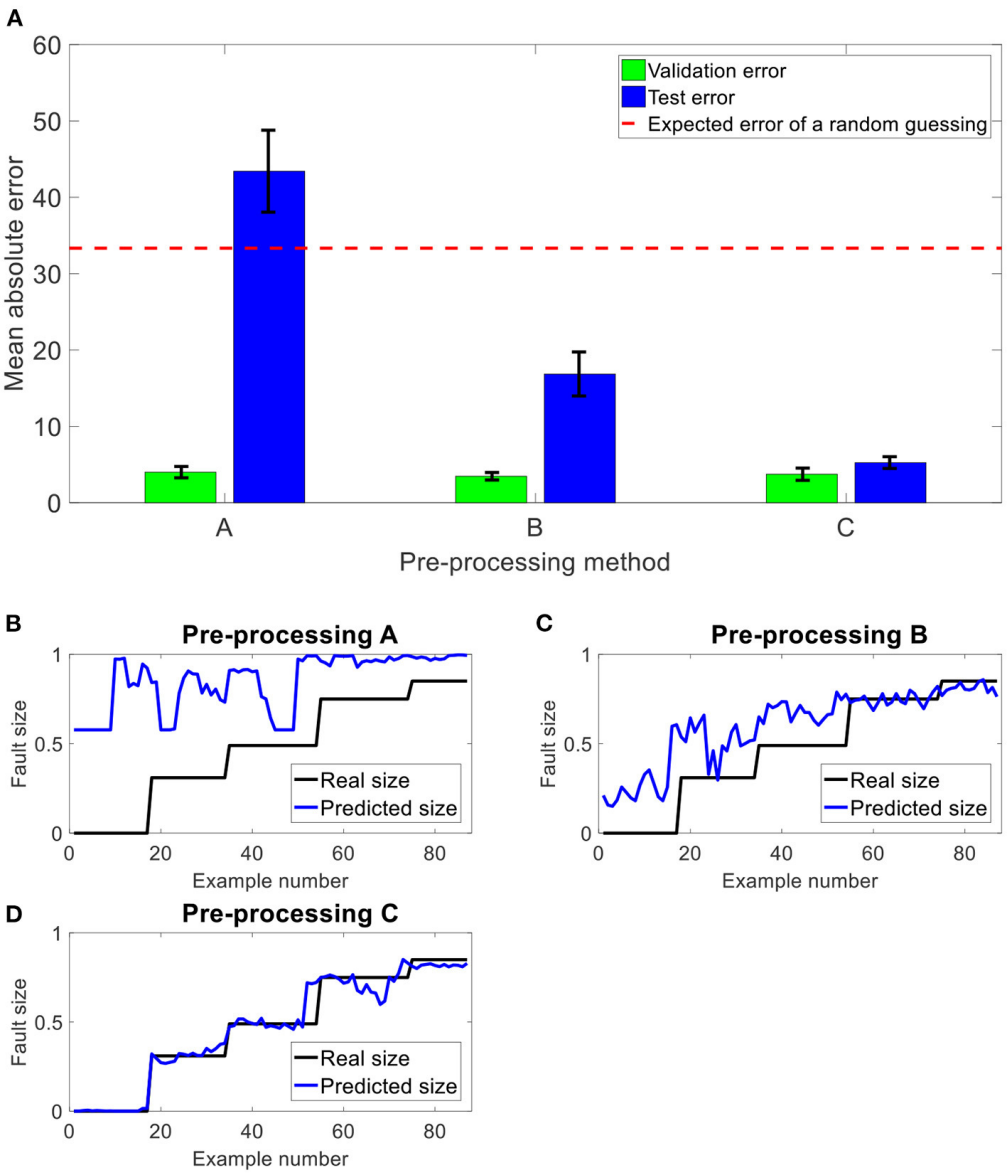
Results of the CNN over three types of pre-processing methods. **(A)** The mean errors and their standard deviation (black) of the CNN over the validation and test sets, repeated 20 times. For each pre-processing method – A, B and C – an example of the predicted and real sizes of the test's signals are presented in **(B–D)**, respectively.

In Figure 9, the results of the CNN over 20 repeated iterations on the validation and test sets are presented for all three pre-processing methods A, B, and C. If the CNN is applied on the test set without mitigating the transfer function effects, the results are worse than random guessing (under the uniform distribution of fault sizes, a random Gaussian leads to a 33% error). The estimation of the transfer function using the minimum phase assumption significantly reduces this error. The estimation of the transfer function by the new technique improves the results by more than a factor of 2, leading to accurate results that are close to the errors over the validation set. The differences between the errors over the validation set and the test set in C can be attributed to the added noise in the generation process of the test set described in Figure 7. The suppression of the transfer function effects using the new technique improves the generalization abilities across the different machines (different by the transfer function), leading to a successful TDM.

During the training phase, the CNN learns several features (Goodfellow et al., 2016) over the vibration signals, enabling successful prediction of the fault size. As a result of the random nature of the learning process of the CNN [random initialization of the weights, random examples of the mini-batch during the stochastic gradient descent (SGD), etc.] (Goodfellow et al., 2016), the CNN could learn different weights in each repeated iteration, leading to different extracted features. Hence, when the transfer function effects are not suppressed (in methods A & B), different features lead to different results because, in some of the iterations, those features are highly affected by the transfer function effects, and in other cases less. We postulate that this phenomenon causes a large standard deviation in A and B. The standard deviation over the test set is smaller in B than in A because some of the effects of the transfer function are mitigated in B as opposed to A.

## Summary

Deep learning is a promising approach for PHM by vibration signals. However, two main challenges complicate the application of deep learning tools for rotating components: (1) the shortcomings in examples of fault signals from real machinery and (2) generalization across different machines (TDM). A digital twin is a promising tool for mitigating the first challenge, while generalizing to new machinery seems still to be a challenge for fault diagnostics.

This paper demonstrates the importance of accounting for the transfer function for TDM, and presents a new approach for estimating the phase of transfer functions for gear signals. The contribution of the new approach for generalization abilities was demonstrated, resulting in an improvement of more than a factor of 3 over the current technique, assuming a minimum phase function for the estimation of the transfer function (from an error of 16.9% in method B in Figure 9A to 5.3% in method C in Figure 9A).

As demonstrated in Figure 9, the ability of this approach to significantly reduce the generalization error between the training and test sets from 13.4% in method B in Figure 9B to 1.5% in method C in Figure 9C significantly improved the result over the signal in the test set (after the transfer function) from 16.9% in method B in Figure 9B to 5.3% in method C in Figure 9C. This procedure can be applied as part of a TDM procedure and can enable generalization from a digital twin or other source of data to new machinery where faulty data are not available. Hence, while digital twins can help to generate a wide set of fault examples for the training phase, suppression of the transfer function effects can help them to generalize better across different machines.

## Data Availability Statement

The raw data supporting the conclusions of this article will be made available by the authors, without undue reservation.

## Author Contributions

OM: conceptualization, methodology, software, validation, formal analysis, investigation, writing—original draft, writing—review, editing, and visualization. JB and RK: conceptualization, methodology, writing—review, editing, and supervision. All authors contributed to the article and approved the submitted version.

## Conflict of Interest

The authors declare that the research was conducted in the absence of any commercial or financial relationships that could be construed as a potential conflict of interest.

## Publisher's Note

All claims expressed in this article are solely those of the authors and do not necessarily represent those of their affiliated organizations, or those of the publisher, the editors and the reviewers. Any product that may be evaluated in this article, or claim that may be made by its manufacturer, is not guaranteed or endorsed by the publisher.

## Abbreviations

ACS: adaptive clutter separation
AR: autoregressive
ARMA: autoregressive moving-average
CNN: convolutional neural network
FTFF: full tooth face fault
MSE: mean squared error
PCHIP: piecewise cubic Hermite interpolating polynomial
PHM: prognostic health monitoring
ReLU: rectified linear unit
RPS: revolutions per second
SGD: stochastic gradient decent
TDM: transfer across different machines.

## References

[B1] Abu-Mahfouz I. A. A. (2005). comparative study of three artificial neural networks for the detection and classification of gear faults. Int. J. Gen. Syst. 34, 261–277. 10.1080/03081070500065726

[B2] Borghesani P. Pennacchi P. Randall R. B. Sawalhi N. Ricci R. (2013). Application of cepstrum pre-whitening for the diagnosis of bearing faults under variable speed conditions. Mech. Syst. Signal. Process. 36, 370–384. 10.1016/j.ymssp.2012.11.001

[B3] Carden E. P. Fanning P. (2004). Vibration based condition monitoring: a review. Struct. Heal. Monit. 3, 355–377. 10.1177/1475921704047500

[B4] Chen J. F. Wang W. M. Huang C. M. (1995). Analysis of an adaptive time-series autoregressive moving-average (ARMA) model for short-term load forecasting. Electr. Power Syst. Res. 34, 187–196. 10.1016/0378-7796(95)00977-1

[B5] Childers D. G. Skinner D. P. Kemerait R. C. (1977). The cepstrum: a guide to processing. IEEE 65, 1428–1443. 10.1109/PROC.1977.10747

[B6] Choi B. (1992). ARMAM Model Identification, 1st Edn. New York, NY: Springer.

[B7] Cunha A. A. M. F. Caetano E. D. S. (2006). Experimental modal analysis of civil engineering structures. Sound Vib. 40, 12–20.

[B8] Dadon I. Koren N. Klein R. Lipsett M. G. Bortman J. (2020). Impact of gear tooth surface quality on detection of local faults. Eng. Fail. Anal. 108, 104291. 10.1016/j.engfailanal.2019.104291

[B9] Goodfellow I. Bengio Y. Courville A. (2016). Deep Learning. Cambridge, MA: MIT Press.

[B10] Gousseau W. Antoni J. Girardin F. Griffaton J. (2016). Analysis of the Rolling Element Bearing Data set of the Center for Intelligent Maintenance Systems of the University of Cincinnati, 13th C. Paris: MFPT.

[B11] Guo L. Lei Y. Xing S. Yan T. Li N. (2019). Deep convolutional transfer learning network: a new method for intelligent fault diagnosis of machines with unlabeled data. IEEE Trans. Ind. Electron. 66, 7316–7325. 10.1109/TIE.2018.2877090

[B12] Jain P. Poon J. Singh J. P. Spanos C. Sanders S. R. Panda S. K. A. (2020). digital twin approach for fault diagnosis in distributed photovoltaic systems. IEEE Trans. Power Electr. 35, 940–956. 10.1109/TPEL.2019.2911594

[B13] Kenett R. S. Bortman J. (2021). The digital twin in Industry 4, 0. A wide-angle perspective. Qual. Reliab. Eng. Int. 37, 1–10. 10.1002/qre.2948

[B14] Klein R. (2017). Comparison of methods for separating vibration sources in rotating machinery. Mech. Syst. Signal. Process. 97, 20–32. 10.1016/j.ymssp.2017.03.040

[B15] Lehmann E. L. Casella G. (2006). Theory of Point Estimation, 2nd Edn. New York, NY: Springer-Verlag.

[B16] Lei Y. (2016). Intelligent Fault Diagnosis and Remaining Useful Life Prediction of Rotating Machinery, 1st Edn. Amsterdam: Elsevier Inc.

[B17] Lei Y. Yang B. Jiang X. Jia F. Nandi A. K. (2020). Applications of machine learning to machine fault diagnosis: a review and roadmap. Mech. Syst. Signal. Process. 138, 106587. 10.1016/j.ymssp.2019.106587

[B18] Lu R. Shahriar M. R. Borghesani P. Randall R. B. Peng Z. (2022). Removal of transfer function effects from transmission error measurements using cepstrum-based operational modal analysis. Mech. Syst. Signal. Process. 165, 108324. 10.1016/j.ymssp.2021.108324

[B19] Madar E. Klein R. Bortman J. (2019). Contribution of dynamic modeling to prognostics of rotating machinery. Mech. Syst. Signal. Process. 123, 496–512. 10.1016/j.ymssp.2019.01.003

[B20] Matania O. Klein R. Bortman J. (2021). Novel approaches for the estimation of the spectrum background for stationary and quasi-stationary signals. Mech. Syst. Signal. Process. 167, 108503. 10.1016/j.ymssp.2021.108503

[B21] Matania O. Klein R. Bortman J. (2022). Algorithms for spectrum background estimation of non-stationary signals. Mech. Syst. Signal. Process. 167, 108551. 10.1016/j.ymssp.2021.108551

[B22] Oppenheim A. V. Schafer R. W. Buck J. R. (1999). Discrete-Time Signal Processing, 2nd Edn. Division of Simon and Schuster One Lake Street Upper Saddle, River, NJ: Prentice-Hall, Inc.

[B23] Peeters C. Guillaume P. Helsen J. (2016). Signal pre-processing using cepstral editing for vibrationbased bearing fault detection. ISMA. 2016, 2489–501.

[B24] Peeters C. Guillaume P. Helsen J. (2017). A comparison of cepstral editing methods as signal pre-processing techniques for vibration-based bearing fault detection. Mech. Syst. Signal. Process. 91, 354–381. 10.1016/j.ymssp.2016.12.036

[B25] Randall R. B. (2004a). State of the art in monitoring rotating machinery - Part 1. J. Sound. Vib. 38, 14–21.

[B26] Randall R. B. (2004b). State of the art in monitoring rotating machinery - Part 2. J. Sound. Vib. 38, 10–7.

[B27] Randall R. B. (2011). Vibration-Based Condition Monitoring – Industrial, Aerospace and Automotive Applications, 1st Edn. Chichester, West Sussex: WILEY.

[B28] Randall R. B. (2017). A. history of cepstrum analysis and its application to mechanical problems. Mech. Syst. Signal. Process. 97, 3–19. 10.1016/j.ymssp.2016.12.026

[B29] Randall R. B. Peeters B. Antoni J. Manzato S. (2012). New cepstral methods of signal pre-processing for operational modal analysis. Int. Conf. Noise. Vib. Eng. 1, 755–764.

[B30] Sawalhi N. Randall R. B. (2005). Spectral Kurtosis Enhancement Using Autoregressive Models. Melbourne: ACAM. 231–6.

[B31] Sawalhi N. Randall R. B. (2013). Localized Fault Detection and Diagnosis in Rolling Element Bearings: A Collection of the State of Art Processing Algorithms. Melbourne: AIAC. 15.

[B32] Shalev-Shwartz S. Ben-David S. (2013). Understanding Machine Learning: From Theory to Algorithms. New York, NY: Cambridge University Press.

[B33] Smith W. A. Randall R. B. (2016). Cepstrum-based operational modal analysis revisited: a discussion on pole–zero models and the regeneration of frequency response functions. Mech. Syst. Signal. Process. 79, 30–46. 10.1016/j.ymssp.2016.02.030

[B34] Wallach D. Goffinet B. (1989). Mean squared error of prediction as a criterion for evaluating and comparing system models. Ecol. Modell. 44, 299–306. 10.1016/0304-3800(89)90035-5

[B35] Xia M. Shao H. Williams D. Lu S. Shu L. de Silva C. L.. (2021). Intelligent fault diagnosis of machinery using digital twin-assisted deep transfer learning. Reliab. Eng. Syst. Saf. 215, 107938. 10.1016/j.ress.2021.107938

[B36] Xu Y. Sun Y. Liu X. Zheng Y. (2019). A digital-twin-assisted fault diagnosis using deep transfer learning. IEEE Access 7, 19990–19999. 10.1109/ACCESS.2018.2890566

[B37] Yang B. Lei Y. Jia F. Xing S. (2019). An intelligent fault diagnosis approach based on transfer learning from laboratory bearings to locomotive bearings. Mech. Syst. Signal. Process. 122, 692–706. 10.1016/j.ymssp.2018.12.051

[B38] Zhang L. Lin J. Liu B. Zhang Z. Yan X. Wei M. (2019). A review on deep learning applications in prognostics and health management. IEEE Access 7, 162415–162438. 10.1109/ACCESS.2019.295098533733218

